# Analyzing Trends in the Incidence of Trauma and Mechanisms of Injury Before, During, and After COVID-19 Lockdown in a Staten Island Level 1 Trauma Center

**DOI:** 10.7759/cureus.108172

**Published:** 2026-05-03

**Authors:** Dorian A Bogdanovski, Vladimir Rubinshteyn, Tara Harrington, Johnathon LeBaron, Douglas Cohen, Paul S Issack, Aditi Basu

**Affiliations:** 1 Surgery, Richmond University Medical Center, Staten Island, USA; 2 General Surgery, Richmond University Medical Center, Staten Island, USA; 3 Trauma Services, Richmond University Medical Center, Staten Island, USA; 4 Emergency Medicine, Richmond University Medical Center, Staten Island, USA; 5 Neurosurgery, Richmond University Medical Center, Staten Island, USA; 6 Orthopedic Surgery, Richmond University Medical Center, Staten Island, USA; 7 Surgery, St. George’s University, New York, USA

**Keywords:** assault-related injuries, covid-19 outbreak, gunshot injuries, level 1 trauma center, major trauma

## Abstract

Background

The COVID-19 pandemic has impacted every branch of medical care. Our level 1 trauma center reported a decrease in the incidence of trauma during the initial months of the pandemic. In this follow-up study, we compared the incidence of trauma in our trauma center during the COVID-19 lockdown in New York City to both pre- and post-lockdown periods.

Methodology

A retrospective review was conducted among all adult patients who presented to the Emergency Department at Richmond University Medical Center in Staten Island, from 2019 through 2023. Data were stratified by time, with strata being pre-lockdown, lockdown (March 2020 through June 2021), and post-lockdown. Mechanism of injury was categorized as either falls, non-fall accidents, traffic-related injuries, including pedestrians and cyclists, and all assaults, including gunshot wounds and stab wounds. Data were also categorized by trauma activation level (partial activation, full activation, consultation, or no activation). Analysis of variance was performed to compare the monthly average of the incidence of trauma during these time periods.

Results

With this more complete data, it was determined that while there was no difference in these time periods in the overall incidence of trauma, there was a significant difference in the incidence of assaults, stabbings, and gunshot wounds, with a significant increase in gunshot wounds during the lockdown period, along with a significant decrease in the incidence of these mechanisms in the post-lockdown period.

Conclusions

While previous data show an overall decrease in the incidence of trauma activations in the early days of the pandemic, this study shows a change in the pattern of mechanisms of injury. Our trauma center may expect and prepare for additional assaults and penetrating injuries during a future epidemic, leading to city-wide lockdown.

## Introduction

The COVID-19 pandemic placed a great strain on healthcare systems and society at large throughout the United States and the world. New York City saw its first case and the beginning of a lockdown in March 2020. Public schools were closed, and non-essential work and travel were restricted. Numerous trauma centers in the United States and abroad noted a decrease in the number of trauma patients they were seeing, likely as a result of these restrictions [1-19]. Retrospective analyses for subspecialties treating isolated injuries also indicated a decrease in their injuries of interest [3,14,15,18,19]. An American retrospective analysis from 2017 to 2020 found a dramatic 57.4% decrease in trauma admissions during the COVID-19 pandemic, with motor vehicle accidents dropping by 80.5% (p < 0.001) following the implementation of stay-at-home orders [2]. A similar national study of trauma patients across 92 centers highlighted a general decline in admissions during COVID-19 surges [7]. Analysis of trauma presentations at a level I trauma center in Georgia showed a decrease in motor vehicle crashes [5]. A study from a trauma center in Boston found a significant decline in nonviolent trauma (from 21.11 to 5.17 activations) but noted stable violent crime rates in vulnerable communities, emphasizing the need for targeted intervention (rate ratio = 0.84) [6]. New York City Health and Hospitals Corporation conducted a study comparing trauma visits from March 2020 through May 2020 against March through May of the three prior years and found a 25% decrease in trauma visits and 50% increase in penetrating injuries during that time [13].

There is a lack of data regarding the time period immediately following the pandemic. A literature review compared the incidence of orthopedic injury before, during, and after the pandemic and concluded that rates of orthopedic injury have returned to pre-pandemic levels [3], but given its scope, it cannot be extrapolated to traumas in general. Our institution performed a study in 2022 comparing the overall incidence and mechanism of injury for pre-pandemic, initial wave, and protracted phase. The study found an increase in gunshot wounds, assault leading to blunt trauma, and motorcycle collisions, as well as a decrease in blunt assaults during the initial wave of COVID-19 [5]. However, the timeline of the study was limited only to the year before the pandemic and the time during lockdown. At the time of the writing of this manuscript, there are no studies from trauma centers comparing the epidemiology of trauma before, during, and after the pandemic.

This study aims to describe the changes in the characteristics of adult trauma visits to a level 1 trauma center in Staten Island before, during, and after the COVID-19 lockdown, particularly the monthly incidences of accidental injuries, traffic injuries, assaults, and penetrating trauma, as well as different levels of trauma activations. By comparing the monthly incidence of traumas over a longer period of time, we aim to control for seasonal effects. An additional goal is to analyze the trends regarding the levels of trauma activations. Ultimately, with a larger pool of patients and a longer period of interest, we hope to be able to determine whether there was a significant difference in trauma visits during these three periods.

## Materials and methods

A retrospective review was conducted among all adult patients who presented to the Emergency Department at Richmond University Medical Center in Staten Island, from 2019 through 2023, who met the criteria for inclusion in the American College of Surgeons National Trauma Databank. Inclusion criteria for this study comprised patients who were seen in the Emergency Department of Richmond University Medical Center, aged 18 years or higher, who were seen in the emergency room as either a trauma code activation or trauma consultation, or admitted to an inpatient service with a traumatic injury. Exclusion criteria comprised patients whose age could not be verified with legal identification, patients who did not have a documented mechanism of injury, and patients who sustained traumatic injury during their hospitalization. The total number of trauma visits each month was tabulated and stratified by mechanism of injury. These monthly incidences were then stratified by whether they occurred in the pre-lockdown, lockdown, and post-lockdown periods. The lockdown period was selected to be from March 2020, the month when New York City public schools were closed, through June 2021, as Mayor de Blasio declared New York City “fully reopened” on July 1st, 2021. For each patient meeting the criteria for the trauma registry, the level of trauma activation and mechanism of injury were reported. For part of the analysis, mechanisms of injury were grouped into the following categories: falls, non-fall accidents, traffic-related injuries, including pedestrians and cyclists, and all assaults, including gunshot wounds and stab wounds. Non-fall accidents included patients who were struck by falling objects, injuries involving animals, sustained sports injuries, suffered machine injuries, sustained burns, inhaled smoke, or suffered electrical injuries. The level of activation was reported as either full, partial, consult, or non-activated. This resulted in a dataset where each month was an independent variable, with dependent variables of total incidence of traumas, incidence of traumas by mechanism, and incidence of traumas by level of activation. JASP 0.19.1 was used for data analysis. Analysis of variance (ANOVA) was conducted to compare the monthly average incidence of each mechanism and the level of activation between the time periods described above.

## Results

A total of 7,520 adult patients met the criteria for inclusion in the trauma registry. Of these patients, 7,430 had a recorded mechanism of injury and trauma activation level and were included in the study. Of these patients, 1,871 presented in the pre-lockdown period, 1,902 presented in the lockdown period, and 3,657 presented in the post-lockdown period. In the pre-lockdown period, there was an average of 133.64 traumas per month (SD = 20.41); there was an average incidence of 118.88 traumas per month in the lockdown period (SD = 21.11); and there was an average of 121.9 traumas per month in the post-lockdown period (SD = 21.01). ANOVA was conducted between the mean monthly trauma activations for each time period. The sum of squares was 1,852.67, with a mean square of 926.34, F-statistic of 2.12, and p-value of 0.129. Residual analysis showed a sum of squares of 24,897.66 and a mean square of 436.80. The demographic characteristics of the study population are presented in Table 1.

**Table 1:** Demographic characteristics of the study population. Demographic information of patients presenting with trauma for the duration of the study. The lowest age was 18.00 years, and the highest age was 107.55 years.

Demographics	
Average age (years ± SD)	59.08 ± 22.02
Males	2,629 (59.94%)
Females	1,757 (40.06%)
Total	4,386

The incidence of falls was analyzed next. The monthly average of falls was 79.36 (SD = 12.43) in the pre-lockdown period, 71.94 (SD = 9.91) in the lockdown period, and 71.87 (SD = 20.46) in the post-lockdown period. ANOVA was conducted between the time periods, showing a sum of squares of 598.32, a mean square of 299.16, an F-statistic of 1.09, and a p-value of 0.343. Residual analysis showed a sum of squares of 15,621.62 with a mean square of 274.06. 

For non-fall accidental injuries, there was an average of 5.64 per month (SD = 2.205) for pre-lockdown, 5.44 per month (SD = 2.80) for lockdown, and 4.67 (SD = 2.869) for post-lockdown. ANOVA showed a sum of squares of 11.58, a mean square of 5.79, an F-statistic of 0.79, and a p-value of 0.460. Residual analysis showed a sum of squares of 419.82 and a mean square of 7.37.

For all assaults, there was an average of 19.07 (SD = 5.85) per month in pre-lockdown, 18.06 (SD = 4.524) per month in lockdown, and 13.73 (SD = 5.88) per month in post-lockdown. ANOVA showed a sum of squares of 353.20, with a mean square of 176.60, an F-statistic of 5.74, and a p-value of 0.005. Residual analysis showed a sum of squares of 1,753.73 with a mean square of 30.77.

For assaults not involving stabbing or gunshot wounds, the monthly averages were 12.07 (SD = 4.63) for pre-lockdown, 8.81 (SD = 3.23) for lockdown, and 8.93 (SD = 4.63) for post-lockdown. ANOVA showed a sum of squares of 108.70, a mean square of 54.35, an F-statistic of 2.93, with a p-value of 0.061. Residual analysis showed a sum of squares of 1,057.23 and a mean square of 18.55.

For assaults involving stabbing, there were 5.50 (SD = 2.77) for pre-lockdown, 6.50 (SD = 2.90) per month for lockdown, and 3.40 (SD = 2.08) per month for post-lockdown. ANOVA showed a statistically significant difference between each period, with a sum of squares of 111.48, a mean square of 55.74, an F-statistic of 9.06, and a p-value <0.001. Residual analysis showed a sum of squares of 350.70 and a mean square of 6.15.

For assaults with gunshot wounds, there were 1.50 (SD = 1.23) per month for pre-lockdown, 2.75 (SD = 1.73) per month for lockdown, and 1.33 (SD = 1.49) per month for post-lockdown. ANOVA revealed a statistically significant difference for these periods, with a sum of squares of 22.08, a mean square of 11.04, an F-statistic of 4.87, and a p-value of 0.011. Residual analysis showed a sum of squares of 129.17 and a mean square of 2.27.

For traffic-related injuries, there was a mean of 23.00 (SD = 6.60) per month for pre-lockdown, 18.69 (SD = 8.08) per month for lockdown, and 24.00 (SD = 9.89) per month for post-lockdown. ANOVA showed a sum of squares of 302.21, a mean square of 151.17, an F-statistic of 1.97, and a p-value of 0.149. Residual analysis showed a sum of squares of 4,379.44 and a mean square of 76.83. Given the apparent difference in incidence of traffic injuries between the time periods without statistical significance, ANOVA was conducted for each individual mechanism of injury, which yielded no statistically significant results (Tables 2, 3).

**Table 2:** Monthly incidence of trauma by mechanism and time period. The average monthly incidence of each category of mechanism for the pre-lockdown, lockdown, and post-lockdown periods. Standard deviation is shown above for each average value.

Monthly incidence of trauma by mechanism and time period	Monthly incidence ± SD		
	Pre-lockdown	Lockdown	Post-lockdown
All traumas	133.64 ± 20.46	118.88 ± 21.11	121.9 ± 21.01
Falls	79.36 ± 12.43	71.94 ± 9.91	71.87 ± 20.46
Accidents (excluding falls)	5.64 ± 2.21	5.44 ± 2.80	4.67 ± 2.87
All assaults	19.07 ± 5.85	18.06 ± 4.52	13.73 ± 5.88
Assaults (excluding stabbing and gunshots)	12.07 ± 4.63	8.81 ± 3.23	8.93 ± 4.63
Stabbing	5.5 ± 2.77	6.5 ± 2.90	3.4 ± 2.08
Gunshot wounds	1.5 ± 1.23	2.75 ± 1.73	1.33 ± 1.49
Traffic injuries	23 ± 6.60	18.69 ± 8.08	24 ± 9.89

**Table 3:** Comparison of monthly incidence of traumas between time periods. Analysis of the incidence of each mechanism between pre-lockdown, lockdown, and post-lockdown time periods using analysis of variance. P-values <0.05 were considered significant.

Category	Sum of squares	Degrees of freedom	Mean square	F	P-value
All traumas	1,852.67	2	926.34	2.12	0.129
Falls	598.32	2	299.16	1.09	0.343
Accidents (excluding falls)	11.58	2	5.79	0.79	0.460
Assaults	353.20	2	176.6	5.74	0.005
Assaults (excluding stabbing or gunshots)	108.70	2	54.35	2.93	0.061
Stabbing	111.48	2	55.74	9.06	<0.001
Gunshot wounds	22.08	2	11.04	4.87	0.011
Traffic injuries	302.21	2	151.11	1.97	0.149

Next, differences in the level of activation were analyzed. ANOVA was conducted for each level of activation. There was a statistically significant difference between the monthly average number of partial activations for each time period, with a sum of squares of 2,507.60, a mean square of 1,253.80, an F-statistic of 4.06, and a p-value of 0.022. There was also a statistically significant difference in the number of trauma consultations, with a sum of squares of 2,385.67, a mean square of 1,192.84, an F-statistic of 16.31, and a p-value <0.001. No statistically significant difference was seen for partial activations or non-activations (Tables 4, 5).

**Table 4:** Monthly incidence of levels of trauma activations. Monthly incidence of each level of trauma activation for pre-lockdown, lockdown, and post-lockdown time periods. “Not levelled” refers to the case where a patient presented without a trauma activation but was found later to have met the criteria for either partial or full activation.

	Monthly incidence ± SD		
	Pre-lockdown	Lockdown	Post-lockdown
Partial activation	84.36 ± 13.44	71.88 ± 15.64	87.17 ± 19.90
Full activation	9.14 ± 4.11	10.19 ± 3.27	8.23 ± 3.52
Consultation	31.36 ± 11.14	27.63 ± 8.69	17.03 ± 7.01
Not leveled	8.79 ± 4.44	9.06 ± 4.11	8.5 ± 6.96

**Table 5:** Comparison of level of activation between time periods. Analysis of the incidence of activation levels between pre-lockdown, lockdown, and post-lockdown time periods using analysis of variance. P-values <0.05 were considered significant.

Activation level	Sum of squares	Degrees of freedom	Mean square	F	P-value
Partial	2,507.60	2	1,253.80	4.09	0.022
Full	40.42	2	20.21	1.56	0.219
Consultations	2,385.67	2	1,192.84	16.31	<0.001
Non-leveled	3.39	2	1.69	0.05	0.951

For each mechanism and level of activation that was found to have a statistically significant difference between time periods, a linear regression model was constructed to compare the effects of each time period on the average incidence of traumas and determine whether the incidence of trauma in the post-lockdown period was comparable to pre-lockdown. For all assaults, there was a statistically significant difference between the coefficient for pre- and post-lockdown (coefficient = -5.338, p = 0.004), with no such difference between pre-lockdown and lockdown (coefficient = -1.009, p = 0.621). There was a similar difference for the stabbings between pre-lockdown and post-lockdown (coefficient = -2.100, p = 0.011), with a non-significant increase from pre-lockdown to lockdown (coefficient = 1.000, p = 0.275). Interestingly, gunshot wounds showed no differences between pre- and post-lockdown (coefficient = -0.167, p = 0.734), but there was a statistically significant difference between pre-lockdown and lockdown (coefficient = 1.250, p = 0.027). 

For partial activations, while there was a statistically significant difference between the three time periods in ANOVA, there was no difference between pre-lockdown and lockdown (coefficient = -12.482, p = 0.056), as well as between pre- and post-lockdown (coefficient = 2.810, p = 0.622). However, for consults, there was a statistically significant difference between pre- and post-lockdown (coefficient = -14.324, p = <0.001).

## Discussion

This analysis found statistically significant differences between pre-lockdown, lockdown, and post-lockdown incidences of all assaults, stabbings, gunshot wounds, partial activations, and consults. The linear regression models were constructed to determine if the average monthly incidence of these mechanisms or activations was similar between pre- and post-lockdown periods, indicating a return to pre-pandemic levels. There was no statistically significant difference in partial trauma activations between pre- and post-lockdown and between pre-lockdown and lockdown, despite a statistically significant difference between all three in ANOVA. We can interpret this as there being a significant variance in the average number of partial activations between the time periods for the duration of the study, but no major differences between pre-lockdown and the other two time periods. For consults, there was a significant decrease when comparing pre-lockdown monthly incidence to post-lockdown, while there was no significant difference between pre-lockdown and lockdown. Thus, we conclude that the post-lockdown period saw a similar incidence of partial trauma activations and a lower incidence of consults, when compared to the pre-lockdown period.

The mechanisms of all assaults, stabbings, and gunshot wounds were found to have a significant difference between the three time periods. Logistic regression modeling showed a significant decrease in the incidence of both stabbings and all assaults for the post-lockdown period compared to the pre-lockdown period. In both cases, there was no significant difference between the pre-lockdown and the lockdown periods. There was also a statistically significant increase in gunshot wounds between the pre-lockdown and lockdown periods for gunshot wounds, with no difference between pre- and post-lockdown periods.

To briefly summarize the above findings, we can conclude that there was a significant difference between the monthly incidence of all assaults, stabbings, gunshot wounds, partial activations, and trauma consultations. In the post-COVID-19 period, there was a significant decrease in the monthly incidence of all assaults, stabbings, and trauma consultations. Meanwhile, the monthly incidence of gunshot wounds in the post-lockdown period returned to a level comparable to the pre-lockdown period, after a significant increase during the lockdown period. The variance of the monthly incidence of partial activations between each time period was significant, but there was no significant difference between pre-lockdown and lockdown, as well as pre-lockdown and post-lockdown.

Overall, there was no statistically significant difference in the total number of trauma activations per month between the pre-lockdown, lockdown, and post-lockdown periods. This is in contrast to the findings of other institutions, which have described a significant decrease in traumas during the COVID-19 pandemic. The majority of traumas that occurred during all three periods were falls, with no significant difference between the mean monthly incidence of all three periods. Because of this, the decision was made to analyze all accidental injuries, excluding falls, under the hypothesis that workplace restrictions may have reduced the incidence of injuries overall. However, there was no statistically significant difference between each period, with each only having an average of about five non-fall accidents per month. The low incidence of non-fall traumas makes more in-depth analysis of this category quite low-powered and unsuitable for drawing conclusions, but one could infer that this may be due to frontline workers continuing to work on-site, representing over 25% of New York City’s workforce) [13,16]. Our institution’s prior study found a significant difference between pre-pandemic and initial wave in strike injuries, but no such difference in the monthly incidence of strike injuries was found when comparing the pre-, post-, and lockdown periods.

In our previous study, there was a significant increase in motorcycle collisions in the initial phase. However, there was no statistically significant difference when comparing pre-, post-, and lockdown periods. This may be due to the prior study’s time stratification, i.e., the initial wave period spanned from March 1, 2020, through September 1, 2020, which encompasses the summer months, a time when one might be more likely to ride a motorcycle. This new analysis focuses on monthly incidences of trauma over a longer period of time to control for any seasonal variance. It may be the case that the prior study’s initial wave period is confounded by the fact that the entire period spans only the spring and summer months.

Further analysis of traffic-related injuries did not reveal any significant differences between the time periods. Originally, it was hypothesized that there would be a significant reduction in traffic injuries in the lockdown and post-lockdown periods, but that was not the case. It would be interesting to determine if there were any changes in the incidence of traffic injuries in other metropolitan hospitals. Compared to other boroughs in New York City, public transit in Staten Island is not as comprehensive, and geography may have played a role in these findings. Areas with more robust public transportation may have seen a change in the incidence of traffic injuries, as there is a reliable alternative to driving.

As mentioned earlier, there was a statistically significant difference between time periods for all assaults, stab injuries, and gunshot wounds. In the case of both stab injuries and gunshot wounds, there was an increase in incidence during the lockdown periods. Interestingly, there was a decrease in all assaults (which was significant) and a decrease in assaults not involving stabbing or shooting (which was not significant). The majority of assaults seen did not involve stabbing or shooting. These figures also correlate with the New York Police Department’s crime statistics for 2020, which showed a 1.5% decrease in assaults, 97% increase in shootings, and 44% increase in murders, though a figure was not offered for stabbings [13,16]. Through our analysis, we noted about 5% decrease in injuries from assault and about 83% increase in injuries from shooting during the lockdown period, which was similar to the figures noted in our prior study. NYC Health + Hospitals also reported an increase of 50% in penetrating injuries [13,16]. Data from the Federal Bureau of Investigation Uniform Crime Reporting Program shows there was an 11.7% increase in assaults, and that 79% of homicides involved a firearm, though a national statistic on shootings or gunshot injuries was not provided, as they are not universally reported.

As with any project, this study is not without its limitations. The patient population was derived from data from only a single center. While the results of the analysis do correlate with data from crime databases, they cannot be used to infer similar conclusions for the entirety of Staten Island. Additionally, only data from 2019 onward was available for the study, and the lockdown period was only 15 months. This limitation of time reduces the size of the study population and, therefore, its power. During the time period of this study, our institution also introduced two new subcategories of partial activation: "Code Hip" for patients suspected of having femoral neck fractures, and “Level G,” as in “geriatric,” for patients aged 65 and older who sustained a low-energy fall. As these categories were not established during the pre-lockdown and lockdown periods, it cannot be ascertained within this analysis whether their implementation affected the overall incidence of trauma activations.

## Conclusions

In the wake of the COVID-19 pandemic, it is important to characterize trends of traumatic injuries at the institutional and local level so that this data may be used to inform resource allocation during any future mass health crisis. While many institutions have analyzed the changes in the utilization of their trauma services, the body of evidence regarding changes in the post-COVID-19 era is relatively slim. The overall experience of our institution did not necessarily align with those of other institutions, namely, that we did not note a decrease in the overall incidence of traumas. Although we had hypothesized there would be a decrease in accidental trauma and traffic injuries, there was no statistically significant change in either category. The increase in gunshot wounds during the lockdown period was consistent with other institutions in New York City. There was significant variance in partial activations, but there was no significant difference between the monthly incidence of partial activations in pre-lockdown compared to either lockdown or post-lockdown. There was a significant decrease in consultations in the post-lockdown period compared to pre-lockdown. In short, our institution’s “new normal” after the lockdown may be fewer assaults, stabbings, and consults, with a return to pre-pandemic numbers of gunshot wounds (after a significant increase) and partial trauma activations. Just as each institution’s patient population is unique, so are the challenges they face during a health crisis, and it is imperative that trauma centers analyze their institutional data to be prepared in the event of another pandemic.
